# i-BLESS is an ultra-sensitive method for detection of DNA double-strand breaks

**DOI:** 10.1038/s42003-018-0165-9

**Published:** 2018-10-31

**Authors:** Anna Biernacka, Yingjie Zhu, Magdalena Skrzypczak, Romain Forey, Benjamin Pardo, Marta Grzelak, Jules Nde, Abhishek Mitra, Andrzej Kudlicki, Nicola Crosetto, Philippe Pasero, Maga Rowicka, Krzysztof Ginalski

**Affiliations:** 1 Laboratory of Bioinformatics and Systems Biology, Centre of New Technologies, University of Warsaw, 02-089 Warsaw, Poland; 2 Department of Biochemistry and Molecular Biology, University of Texas Medical Branch at Galveston, Galveston, TX 77555 USA; 3 Institut de Génétique Humaine, CNRS, Université de Montpellier, 34396 Montpellier, France; 4 Institute for Translational Sciences, University of Texas Medical Branch at Galveston, Galveston, TX 77555 USA; 5 Sealy Center for Molecular Medicine, University of Texas Medical Branch at Galveston, Galveston, TX 77555 USA; 6 Sealy Center for Structural Biology and Molecular Biophysics, University of Texas Medical Branch at Galveston, Galveston, TX 77555 USA; 7 Science for Life Laboratory, Department of Medical Biochemistry and Biophysics, Karolinska Institutet, Stockholm, SE-17165 Sweden

## Abstract

Maintenance of genome stability is a key issue for cell fate that could be compromised by chromosome deletions and translocations caused by DNA double-strand breaks (DSBs). Thus development of precise and sensitive tools for DSBs labeling is of great importance for understanding mechanisms of DSB formation, their sensing and repair. Until now there has been no high resolution and specific DSB detection technique that would be applicable to any cells regardless of their size. Here, we present i-BLESS, a universal method for direct genome-wide DNA double-strand break labeling in cells immobilized in agarose beads. i-BLESS has three key advantages: it is the only unbiased method applicable to yeast, achieves a sensitivity of one break at a given position in 100,000 cells, and eliminates background noise while still allowing for fixation of samples. The method allows detection of ultra-rare breaks such as those forming spontaneously at G-quadruplexes.

## Abstract (web summary)

Anna Biernacka, Yingjie Zhu et al. present i-BLESS, a universal method for detecting genome-wide DNA double strand breaks, optimized here for yeast. By immobilizing cells on agarose beads, the authors are able to achieve efficient diffusion of reagents and labeling of double strand breaks, including ultra-rare breaks such as those at G-quadruplexes.

## Introduction

DNA double-strand breaks (DSBs) are one of the most lethal types of DNA lesions^1^, being a primary source of chromosome translocations and deletions^2^. Since DSBs are the driving force of genomic instability^3^, a hallmark of most cancers^4^, better understanding of genome sensitivity to DSBs and the mechanisms of their formation is essential. In yeast, chromatin immunoprecipitation with antibody against phosphorylated histone H2A (γ-H2A) has been commonly used to map break sites^5^. This method has, however, several disadvantages, in particular γ-H2A does not mark DSBs exclusively^6^ and extends several kilobases away from breaks^7^. Recently, a new method called Break-seq has been proposed to study DSBs in *Saccharomyces cerevisiae*^8^. However, it can only detect DNA ends with 5′ overhangs, which limits its applications. Since *S. cerevisiae* is a premier model for eukaryotic cell biology, functional genomics and systems biology, developing a method for precise DSB detection in yeast is of high importance.

Several next-generation sequencing methods have been recently developed to label DSBs directly and genome-wide in mammalian cells^9–11^, starting with our BLESS (Breaks Labeling, Enrichment on Streptavidin and next-generation Sequencing) method^12^. However, these techniques cannot be applied to detect DSBs in yeast. For instance, BLESS and DSBCapture^9^ employ multiple low-speed (200*g*) centrifugation steps to collect nuclei. Yeast nuclei, due to their very small diameter (2 µm), must be collected using high-speed centrifugations (>4000*g*), which could result in chromatin shearing. One commonly used strategy to overcome this issue is encapsulation of cells in agarose, which protects DNA from mechanical damage. This approach was used by Mimitou et al. in S1-Seq^13^ to label DSBs resulting from end resection in yeast. However, the S1 nuclease used for ends blunting in S1-Seq can also transform single-stranded regions in duplex DNA into DSBs^14^, leading to unspecific labeling. END-seq^10^, another BLESS-based method, employs agarose plugs and is therefore in principle applicable to small cells, but has not been optimized for *S. cerevisiae*. Yeast cells are protected by a thick cell wall, which requires an additional step of enzymatic digestion prior to labeling.

Here, we present i-BLESS (immobilized-BLESS), a new method for direct in situ genome-wide DSB labeling in agarose beads, optimized for yeast, but in principle applicable to all (particularly small) cells. High resolution and sensitivity of i-BLESS allowed us to detect ultra-rare breaks such as off-target locations of endonucleases cleavage and DSBs forming spontaneously at G-quadruplexes.

## Results

### i-BLESS method

**Fig. 1:**
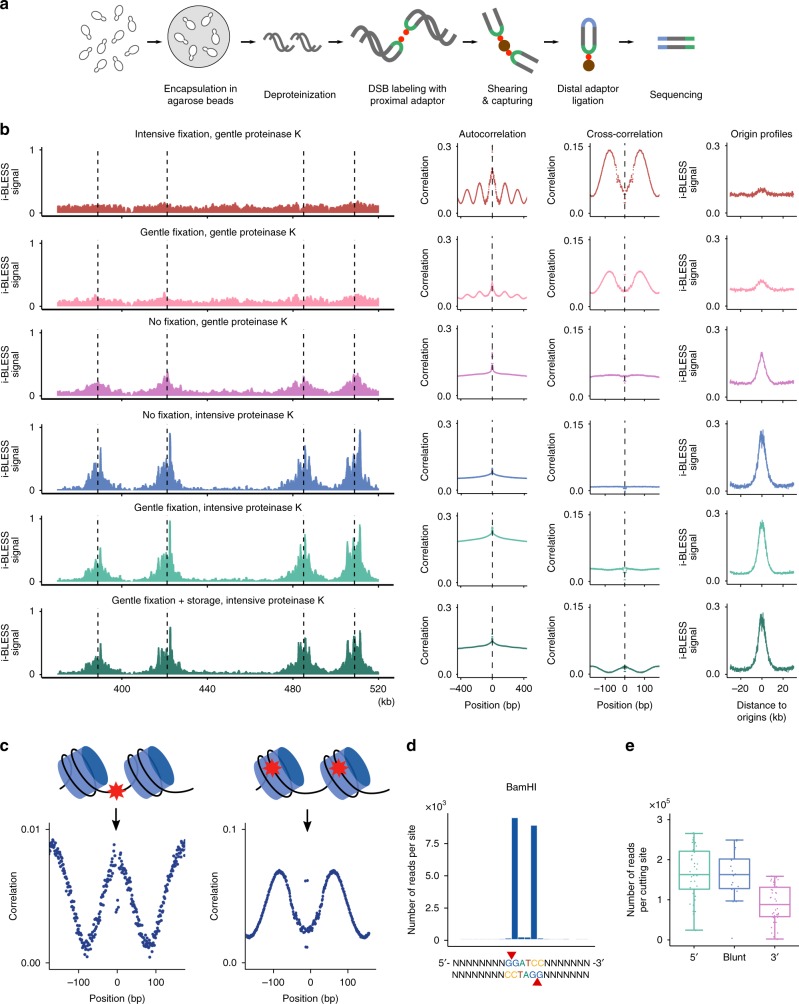
i-BLESS method and its validation. **a** i-BLESS workflow. Briefly, cells are encapsulated in agarose beads, lysed and deproteinated, DSBs are labeled with a biotinylated adapter (proximal) and captured on streptavidin. Free ends of DNA fragments are ligated to the second adapter (distal), and the resulting fragments are amplified and sequenced. **b** Impact of experimental protocol parameters on quality of i-BLESS data. *mec1-1 sml1-1* cells were treated with hydroxyurea and subjected to indicated treatments: intensive fixation: cell fixation with 2% formaldehyde for 30 min; gentle fixation: cell fixation with 2% formaldehyde for 5 min; storage: storage of fixed cells for 7 days at 4 °C; intensive proteinase K: 50 µg mL^−1^ overnight at 50 °C; and gentle proteinase K: 1 µg mL^−1^ for 5 min at 37 °C. For each sample, i-BLESS signal around replication origins (dotted vertical lines) in a representative region of chromosome VII, autocorrelation of i-BLESS signal, cross-correlation of i-BLESS data with MNase-seq data^18^ and averaged i-BLESS signal around replication origins are shown. i-BLESS data in the top two panels, for which signal-to-noise ratio is the lowest (as illustrated by averaged meta-profiles of i-BLESS signal around replication origins), shows clear periodicity in autocorrelation pattern related to nucleosome spacing, suggesting over-fixation as a main source of noise during DSB detection. Reads were normalized to 1 million total reads. **c** Cross-correlation of i-BLESS data with nucleosome positioning data (MNase-seq) characteristic for DSBs located preferentially between nucleosomes (left) or within nucleosomes (right). As MNase signal is increased in nucleosome depleted regions, a peak for cross-correlation observed at position 0 bp (left panel) implies DSBs enriched between nucleosomes, while peaks observed at positions +/−80 bp (right panel) indicate DSBs enriched within nucleosomes. **d** Averaged i-BLESS signal in a 22 bp window around BamHI cutting sites (marked with red arrows). **e** Number of i-BLESS reads at NotI (5′ overhangs), SrfI (blunt ends) and AsiSI (3′ overhangs) recognition sites in wild type cells treated with all 3 enzymes simultaneously. Median (center line), lower/upper quartiles (box limits), and lower/upper adjacent (whiskers) are shown

In i-BLESS cells are embedded in agarose beads (which enables more efficient diffusion of the reagents compared to agarose plugs) using a modified Overhauser’s protocol^15^, followed by spheroplasting, lysis and protein removal (Fig. 1a). Next, similarly as in the original BLESS protocol, the dsDNA ends are blunted, 5′-phosphorylated and ligated with a biotinylated adapter (proximal)^12^. DNA is isolated from agarose, fragmented, and the biotinylated fragments are captured on streptavidin beads. The free ends of the captured fragments are then ligated to a second adapter (distal), and the resulting DNA is linearized, amplified by PCR, and sequenced^16^.

To increase the sensitivity of i-BLESS, we comprehensively analyzed the nature of noise in the data and the impact of varying experimental parameters (fixation duration and proteinase K incubation conditions) on the quality of the results. We computationally analyzed patterns of DSBs detected by i-BLESS to find signatures distinguishing genuine breaks from artifacts and observed a high periodicity of the background signal, with a period of 162 bp, which corresponds to the typical distance between nucleosomes in *S. cerevisiae*^17^ (Supplementary Fig. 1a). Consequently, we compared i-BLESS results with data from MNase-seq^18^, which combines micrococcal nuclease (MNase) digestion and sequencing to determine the approximate genomic positions of nucleosomes. Using cross-correlation analysis, we discovered that in samples exhibiting high-noise the breaks were enriched at positions overlapping with nucleosomes, while in samples showing low-noise the breaks did not present dependence on nucleosome locations or were preferentially located between nucleosomes, as expected (Fig. 1b,c). This observation that artifactual breaks (noise) seem to colocalize with nucleosomes led us to hypothesize that they may be related to over-fixation and incomplete protein removal (Fig. 1b) and that cross-correlation with nucleosome positions can be used to assess quality of the data (Fig. 1c). Indeed, after comprehensive studies, we identified a range of parameters optimal for highly specific DSB detection, among which intensive proteinase K treatment (50 µg mL^−1^ overnight at 50 °C) turned out to be crucial (Fig. 1b). As it was reported that proteinase K treatment at 50 °C might result in the conversion of damaged bases or abasic sites into DSBs, while at lower temperature (30 °C) formation of these artifactual DSBs is considerably reduced^19^, we compared i-BLESS signal for samples subjected to proteinase K digestion at 30 °C and 50 °C. We obtained similar results for both conditions, however, the background signal was slightly higher for samples treated at 30 °C (Supplementary Fig. 1b-c). We therefore recommend using lower temperature during incubation with proteinase K when high levels of abasic sites are expected, e.g., for samples treated with methyl methanesulfonate. The DSB signals obtained from both non-fixed and gently fixed samples, treated with a high dose of proteinase K (50 µg mL^−1^ overnight at 50 °C), were similar and showed very low noise level (Fig. 1b). Moreover, storage of gently fixed samples for one week had no influence on data quality (Fig. 1b). Consequently, in contrast to END-seq^10^, our protocol allows gentle cell fixation without compromising data quality, which is essential when DSB labeling is not possible immediately after induction of DSBs.

### i-BLESS validation

We tested our optimized protocol by introducing DSBs in vitro using BamHI cleavage in G1-arrested wild type yeast cells. No DNA was recovered when either no biotinylated adapter or no T4 DNA ligase were used during the in situ ligation (Supplementary Fig. 1d), demonstrating high specificity of DSB labeling. Sequencing data showed that 99.1% of i-BLESS barcoded fragments contained both proximal and distal barcodes, as intended, while 0.7% exhibited two proximal barcodes (which may correspond to nearby DSBs). 97.9% of all barcoded reads were detected precisely at cutting sites, while 99.6% were detected within +/−1 nt of BamHI recognition sites, proving high specificity of our method (Fig. 1d) with maximal false discovery rate of 0.004 artifactual DSBs for each detected DSB. Of 1667 BamHI sites present in the genome of the yeast strain used in this study, i-BLESS detected 1620 sites. The 47 sites undetected by our method were located in unmappable regions of the genome, meaning that i-BLESS achieved the maximum possible detectability of BamHI sites, irrespective of the nearby GC content. To test further if all kinds of dsDNA ends are detected by i-BLESS efficiently, we labeled DSBs introduced by NotI, AsiSI and SrfI restriction enzymes, which create 5′ overhangs, 3′ overhangs and blunt ends, respectively. While lower amount of reads were present at AsiSI sites, a strong signal was observed for all types of DSBs (Fig. 1e, Supplementary Fig. 1e).

### Comparison with Break-seq

**Fig. 2:**
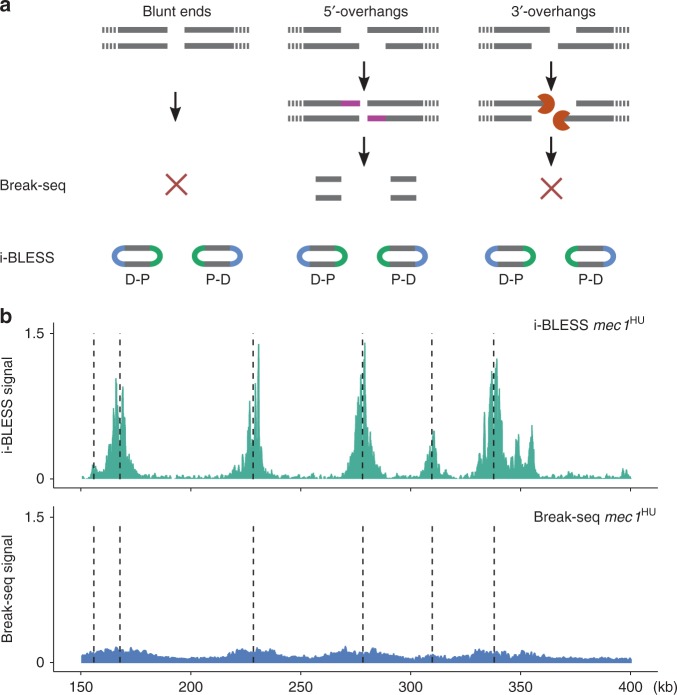
Comparison of i-BLESS and Break-seq. **a** Design of Break-seq renders it unable to detect blunt ends and 3′-overhangs. P and D correspond to proximal and distal adapters, respectively. **b** i-BLESS and Break-seq signals around early replication origins (dotted vertical lines) in a representative region of chromosome XV for HU treated *mec1-1 sml1-1* cells. Reads were normalized to 1 million total reads

Break-seq, a recently developed genome-wide DSB detection method specifically tailored for yeast, relies on DSB labeling in agarose plugs using biotinylated dATPs during blunting, followed by isolation of labeled fragments on streptavidin beads and ligation of Illumina adapters^8^. As during the blunting reaction nucleotides are incorporated only at 5′overhangs, while 3′ overhangs are shortened, blunt ends and 3′overhangs cannot be detected by Break-seq (Fig. 2a). Moreover, information about which end of each DNA fragment corresponds to the original DSB is lost during sample preparation (Fig. 2a). To assess i-BLESS performance relative to Break-seq, we compared the ability of both methods to detect DSBs created by BamHI cutting, which results in 5′overhangs. Our results show that i-BLESS detected 97.2% (1620 out of 1667) BamHI recognition sites, in comparison to 77.2% (1296 out of 1679) sites identified by Break-seq^8^. As opposed to i-BLESS, in Break-seq only 37.9 % of all mapped reads were observed precisely at the expected position. In addition, signal resulting from distal ends of the labeled fragments spread approximately +/−800 bp around BamHI recognition sites (Supplementary Fig. 2). Break-seq inability to distinguish between proximal and distal ends of labeled fragments therefore results in poor spatial resolution of several hundred bp, as compared with single-nucleotide resolution of i-BLESS.

Lack of ability to detect 3′overhangs and blunt ends greatly limits application of Break-seq, what is clearly demonstrated in results obtained for hydroxyurea (HU) treated *mec1-1 sml1-1* cells^8^. Under HU treatment replication forks stall and eventually collapse, resulting in DSBs formation. All homologous repair intermediates and other important break types, e.g., those originated from Okazaki fragments, would manifest as 3′ overhangs and as such would be undetectable by Break-seq. While Break-seq and i-BLESS both detected DSBs accumulated around replication origins during HU exposure, in fact the i-BLESS signal-to-noise ratio was an order of magnitude stronger (Fig. 2b). Break-seq design resulted in loss of majority of HU-induced DSB signal indicating that this method is not optimal to study DSBs occurring in living cells, which could be subjected to resection.

### i-BLESS sensitivity

**Fig. 3:**
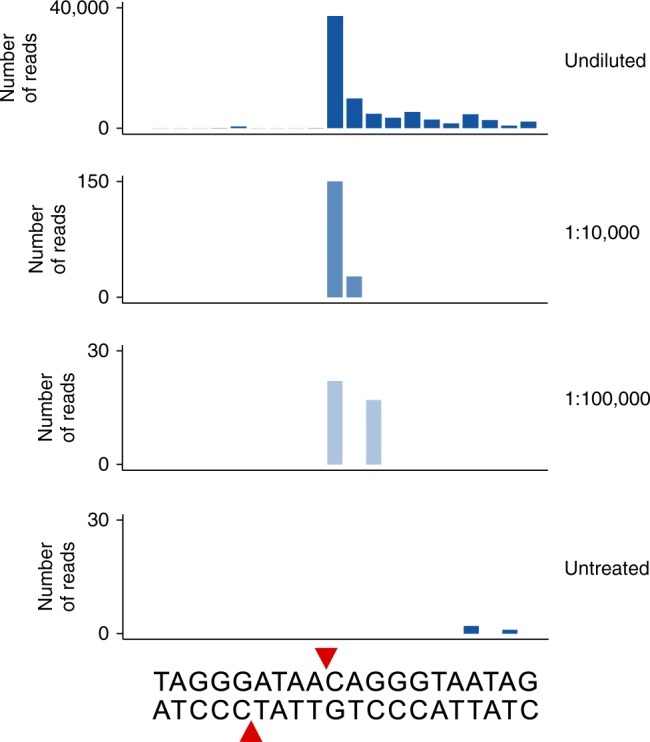
i-BLESS high sensitivity allows detection of ultra-rare breaks. i-BLESS signal around I-SceI recognition site (red arrows) in: non-diluted in vivo Gal treated YBP-275 cells, in vitro I-SceI treated YBP-275 cells mixed with wild type cells in ratios of 1:10,000 and 1:100,000, and untreated YBP-275 cells

Recently, END-seq was shown to be more sensitive than the original BLESS protocol and to be able to detect approximately one induced DSB in 10,000 cells^10^. To test the sensitivity of i-BLESS, we used the YBP-275 yeast strain with Gal inducible I-SceI endonuclease and a single I-SceI recognition site introduced at the ADH4 locus on chromosome VII (as confirmed by analysis of paired-end gDNA sequencing data, Supplementary Table 1). As a benchmark, we labeled in vivo I-SceI induced breaks in undiluted conditions and observed a strong increase of reads mapped to the cutting site upon DSB induction, as compared to untreated cells (Fig. 3). The i-BLESS signal was restricted to one side of the cutting site only, due to a long poly-A region close to the I-SceI site, which interfered with DNA products amplification and mapping. As opposed to experiments performed with in vitro enzymes digestion, the signal was observed not only in direct vicinity of the cutting site, but also within 100 nt from the break site, probably due to its resection. Then, we introduced DSBs in vitro by I-SceI cutting in YBP-275 cells mixed with wild type cells in ratios of 1:10,000 and 1:100,000 (respectively 20,000 and 2,000 cells with DSB among 20 million cells without break at I-SceI site). While a high peak was observed at 1:10,000 dilution, the signal remained detectable even at 1:100,000 dilution, a sensitivity not ever reported before (Fig. 3, Supplementary Fig. 3a). The ability to detect a single DSB per 100,000 cells (providing that at least 2,000 cells with a break at a given position are used) makes i-BLESS the most sensitive DSB detection method published so far. Moreover, in agreement with previous findings that I-SceI can induce breaks at sites that differ from its recognition sequence^20,21^, we observed an increased signal at 24 genomic locations, that shared 10–15 bp identity with the canonical I-SceI sequence (Supplementary Fig. 3b, Supplementary Table 2). None of these sequences was previously reported as a I-SceI-recognized site and only 8 of them agreed with 11-bp invariant I-SceI sequence motif proposed by Petek et al.^20^, proving that actual I-SceI recognition sequence is in fact more diverse than expected. The percentage of cells exhibiting breaks at a given non-canonical I-SceI site varied from 0.01% to 8.29% (estimated by our qDSB-seq approach^22^ to quantify DSBs, see Methods) (Supplementary Table 2), which indicates a potential application of i-BLESS in identifying off-target locations of endonucleases cleavage, even those with very low cutting frequency.

### Detection of rare DSBs at G-quadruplex motifs

**Fig. 4:**
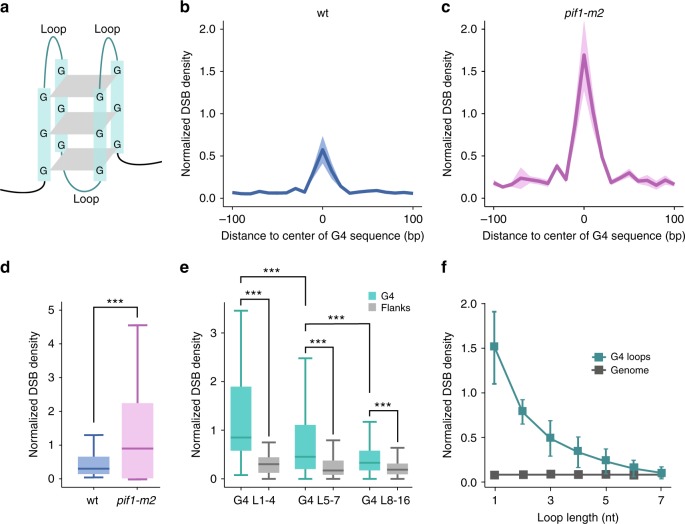
G4-related genome instability. **a** Schematic representation of G4 structure. **b,c** Averaged i-BLESS signal in a 200 bp window centered on G4s, calculated by aligning all G4 centers and using 10 bp bins for wild type and *pif1-m2* cells. Mean values (solid line) and standard deviation (color shade) for three biological replicates are shown. **d** DSB densities inside G4 sequences for wild type (blue, wt) and *pif1-m2* (purple) cells. DSB density was defined as a number of i-BLESS reads mapped to a given region, divided by region length. Median (center line), lower/upper quartiles (box limits), and lower/upper adjacent (whiskers) are shown. *P* value was calculated by paired Wilcoxon signed-rank test, ***P < 0.001. **e** DSB densities inside and outside of G4s containing loops of the indicated length. G4: canonical G-quadruplex structures identified by AllQuad software (see Methods). Flanks: left and right adjacent regions half of the length of their corresponding G4. G4 sequences are classified into: G4 L_1–4_: all loops ≤ 4 nt; G4 L_5–7_: all loops ≤ 7 nt, but at least one loop > 4 nt; G4 L_8–16_: all loops ≤ 16 nt, but at least one loop > 7 nt. Median (center line), lower/upper quartiles (box limits), and lower/upper adjacent (whiskers) are shown. *P* values were calculated by two-sided Kolmogorov-Smirnov test, ***P < 0.001. **f** Average DSB densities for G4 loops of length varying from 1 to 7 nucleotides and control genomic regions of the same length. The 1–7 nt-long control genomic regions were randomly selected. Mean values and standard deviation for three biological replicates are shown. The number of reads were normalized to the total mapped reads to compare DSB densities between replicates

Due to endogenous metabolic reactions and replication stress, hundreds to thousands DNA lesions, such as single-strand breaks or damage to DNA bases, occur in a typical cell each day^23^. DSBs, however, are much more uncommon, as it was estimated that around one spontaneous DSB per cell arises every 4–5 yeast cell division cycles^24,25^. i-BLESS high sensitivity enabled us to study rare DSBs occurring spontaneously in physiological conditions. Specifically, we analyzed the relationship between genome instability and the formation of G-quadruplexes (G4s) in vivo. G4s (Fig. 4a) have been proposed to be involved in the regulation of DNA replication, gene expression and telomere maintenance^26^. They are also considered a possible source of DNA damage, including gross chromosomal rearrangements. G4-related genomic instability is observed typically only upon G4 stabilization^27^, however, using i-BLESS, we detected DSBs at G4 forming sequences in untreated wild type cells. Using qDSB-seq approach, we estimated that the frequency of such breaks in the vicinity of G4s was very low, of the order of 4 DSBs per 1,000 cells for all G4s, and 2 DSBs in 100,000 cells at individual loci, making them detectable only thanks to the very high sensitivity of our method. Nevertheless, we observed a remarkable, 26-fold enrichment of G4s in DSB-rich regions in untreated cells. The increased break signal was higher within G4 sequences (Fig. 4b), as compared to their vicinity, providing clues to possible mechanisms of DSBs formation. To verify that DSBs at these sites are related to G4 structures, we tested if lack of Pif1, a 5′-3′ DNA helicase which binds and unwinds G4 structures in vitro^28^, would affect the number of DSBs at G4 motifs. We labeled breaks in *pif1-m2* mutant cells, which express only the mitochondrial isoform of Pif1 but are defective in its nuclear function. We observed a significant increase of DSB signal (*P* value < 2.2 × 10^−16^, paired Wilcoxon signed-rank test) within G4-forming sequences (Fig. 4b-d), proving that observed DSBs are in fact related to G4s. In addition, while the majority of studies has focused so far on intrastrand G4s^29^, we found that both inter- and intrastrand G4s were equally prone to breakage, as there was no significant difference in break level detected within both G4s configurations (*P* value = 0.38, two-sided Kolmogorov–Smirnov test), indicating the biological relevance of interstrand G4s (Supplementary Fig. 4a).

Typically, G4 structure consists of four tracts of 3 guanines forming guanine tetrads, separated by loops of different lengths (Fig. 4a). While it was previously shown that G4 stability under high salt concentrations decreases with the increasing length of the loops between guanine tracts^30^, it is not known whether this is true for physiological conditions as well. It was also proposed that only G4s with short loops (≤4) can trigger genomic instability^31^, but until now no thorough analysis of the relationship between loop length and the G4-related breaks was conducted. Therefore, we compared DSB densities in G4s divided into subcategories based on the loop length ranging from 1 to 16 nt (see Methods). As expected, we observed the highest DSB densities for G4s with short loops (Fig. 4e, Supplementary Fig. 4b). However, the signal inside G4 was significantly increased (*P* value < 0.001, Wilcoxon signed-rank test) as compared with the flanking regions of the same length for all G4 groups, irrespective of the loop length. These results therefore indicate that G4 motifs containing much longer loops than previously thought, up to 16 nucleotides, can cause genomic instability. This is particularly important, as most computational approaches predict only canonical G4s (G_3–5_N_1–7_G_3–5_N_1–7_G_3–5_N_1–7_G_3–5_), despite the growing evidence, supported by our results, that G4s with longer loops are biologically relevant as well^32^. Also, taking advantage of single-nucleotide resolution of our labeling method, we were able to determine precisely in which part of G4 motif DSBs occur most frequently. We analyzed how loop breakability changes with its length and observed monotonic decrease of loops fragility with their increasing length (Fig. 4f). Short loops are therefore not only responsible for G4s overall fragility, but are also the preferred sites of G4 breakage. However, further studies will be required to elucidate the detailed mechanisms responsible for G4-related DSBs. Recent research indicates that they may be implicated in transcriptional and epigenetic reprogramming^33^. Indeed, while all promoters were significantly enriched in DSBs (*P* value < 0.001, permutation test), we observed more than two-fold higher DSB enrichment in promoters containing G4s than in non-G4 promoters (Supplementary Fig. 4c).

## Discussion

In conclusion, we have developed i-BLESS, a highly sensitive and specific method for precise genome-wide DSB detection, generally applicable to all organisms, here optimized for yeast cells. Due to many advantages, above all easy genetic manipulation, *S. cerevisiae* has been widely used as a model system in almost every area of cell biology, what results in the availability of vast amount of data, from the characterization of genes and proteins to genome-wide comprehensive analyses. Although the yeast genome is around 260 times smaller than the human genome, many of the genes involved in cell cycle regulation in yeast have homologs in human, and 20% of human disease genes have functional equivalents in yeast^34,35^. Thus *S. cerevisiae* can be used to study both elementary cellular processes, as well as mechanisms involved in genomic instability, a hallmark of many human diseases^36–38^. A method for precise DSB detection in yeast is therefore of great importance for studies of mechanisms of DNA damage formation, sensing and repair, what should bring a great advantage to these fields. As techniques used currently for genome-wide labeling of breaks in yeast, such as ChIP-seq for γ-H2A or Break-seq, do not label DSBs specifically or are unable to detect all types of DSBs, i-BLESS is currently the only method that allows precise, specific and highly sensitive detection of DSBs in yeast.

Thorough optimization of i-BLESS procedure enabled us to substantially reduce noise levels, while still permitting fixation. This allows prolonged storage or shipping of samples, which is essential for collaborative projects, e.g., when complex synchronization procedures or exposure to drugs under specific conditions are used. Noise reduction resulted in very high sensitivity of our method, enabling the detection of a single DSB at a given position in 100,000 cells, thus opening up new opportunities to study rare DSBs in physiological conditions. In particular, using i-BLESS we detected a strong enrichment of DSBs at G4-forming sequences in unperturbed wild type yeast cells. We also discovered 24 previously unreported non-canonical I-SceI recognition sites, some of which were cut in only 0.01% of cells. These results indicate a potential role of i-BLESS in identifying off-target locations of endonucleases cleavage, such as those created by CRISPR/Cas9 nucleases. Numerous studies have raised high hopes for CRISPR/Cas9-mediated gene therapy, however off-target activity causing insertions or deletions has been reported to be a common issue for this system^39^. Potential therapeutic application of this technology therefore requires extremely sensitive methods like i-BLESS for testing the specificity of engineered nucleases.

As demonstrated for DSBs created by various restriction enzymes, i-BLESS detects breaks with single-nucleotide resolution. However, DSBs that occur in living cells can be subjected to resection, which creates long (even up to 10 kb^40^) 3′ overhangs. Blunting of such breaks conducted during i-BLESS procedure results in their shortening, which in turn leads to detection of signal in considerable distance from original break site. This problem might be overcome by application of computational modeling of resection or employment of strains deficient in proteins crucial for this process.

Another limitation of i-BLESS is the relatively high number of cells required for the procedure. For encapsulation in agarose beads it is recommended to use 2.5 × 10^9^ yeast cells, for bigger cells smaller amounts can be exploited (i.e. 10^7^ human cells according to the Overhauser’s protocol^15^). In case of yeast, obtaining high amount of material is relatively easy, nevertheless for some experiments, e.g. time-courses, it might be challenging. However, for samples derived from environment or patients, the requirement for high amount of cells would be a major obstacle and would require scaling down the agarose beads encapsulation procedure. It should be also noted that we recommend to use at least 1 μg of DNA for ligation of distal linker and subsequent steps to ensure sufficient amount of output material for representative, good quality libraries for DNA sequencing.

Summarizing, i-BLESS is an innovative and powerful tool to study DNA damage and repair, its unprecedented sensitivity allows detection of DSBs occurring with average frequency as low as 1 in 100,000 cells (providing that at least 2,000 cells with a break at a given position are used). i-BLESS also offers single-nucleotide resolution, ensures specific labeling by employing barcodes and is in general applicable to any organism.

## Methods

### Strains and growth conditions

Yeast strains used in this study are listed in Supplementary Table 3. Cells were grown in YPD medium (BD BactoYeast Extract 1%, BD BactoPeptone 2%, Dextrose 2%) at 25 °C until early log phase and then arrested in G1 for 170 min with 8 μg mL^−1^ α-factor. YBP-275 strain was cultured in YPR medium (BD BactoYeast Extract 1%, BD BactoPeptone 2%, Raffinose 2%), galactose was added for 2 h to induce I-SceI cutting. *mec1-1 sml1-1* cells were released from G1 arrest by addition of 75 µg mL^−1^ Pronase (Sigma). 200 mM HU (Abcam) was added 20 min before Pronase release followed by 1 h incubation. Collected cells were washed with cold SE buffer (5 M NaCl, 500 mM EDTA, pH 7.5) and subjected to DSB labeling with i-BLESS.

### i-BLESS labeling

Approximately 2.5 × 10^9^ yeast cells were resuspended in 5 mL SE buffer and mixed with 5 mL 1% Reducta agarose (Promega) in SE buffer at 40 °C. Cell suspension was mixed with 20 mL liquid paraffin (Merck Millipore) at 40 °C and vigorously shaken by hand for 1 min, until emulsion was formed. The emulsion was then poured into 200 mL ice-cold SE buffer and the mixture was stirred for several minutes. Agarose bead suspension was gently centrifuged (200*g*, 10 min), paraffin layer was removed and agarose bead pellet was washed 3 times with TE buffer. 0.5 mL β-mercaptoethanol, 20 µL of 200 U µL^−1^ lyticase solution (Sigma) and SE to a final volume of 10 mL was then added to the bead pellet, followed by 1 h incubation at 30 °C. Beads were washed with ES buffer (1% sarkosyl, 25 mM EDTA, pH 8.0), resuspended in ES with 50 µg mL^−1^ proteinase K (Sigma) and incubated overnight at 50 °C. After incubation, the beads were washed with TE + 0.1 mM PMSF and twice with TE. For samples treated with restriction enzymes, the beads were washed with appropriate buffer (FastDigest buffer (Thermo Scientific) or CutSmart buffer (NEB)) followed by 1 h treatment with restriction enzymes (BamHI (Thermo Scientific), NotI (NEB), SrfI (NEB), AsiSI (NEB) or I-SceI (NEB)) at 37 °C. Next, the beads were washed in 1× Blunting Buffer (NEB), followed by DNA ends blunting using Quick Blunting kit (NEB) for 2 h. The beads were subsequently washed with T4 ligation buffer and then resuspended in T4 ligation buffer with 100 nM proximal adapter (Supplementary Table 4). After 2 h, T4 ligase was added and the beads were incubated for up to 2 days at 16 °C. After ligation, the beads were washed once with TE, and encapsulated DNA was initially sonicated using Covaris S220. Total DNA was isolated using Zymoclean™ Large Fragment DNA Recovery Kit (Zymo Research) and once again fragmented by sonication to create ~400 bp fragments. Labeled fragments were captured by streptavidin beads (Invitrogen), blunted and phosphorylated using Quick Blunting Kit (NEB), then ligated to a distal adapter (Supplementary Table 4; both proximal and distal adapters are identical to those used in the original BLESS method^12^). The resulting circular DNA was then linearized by I-SceI (NEB) digestion and amplified by PCR. Purified PCR products were subsequently treated with XhoI (NEB) to cleave terminal I-SceI sequences derived from adapters.

### Library preparation and sequencing

Sequencing libraries for i-BLESS and respective gDNA samples were prepared using ThruPLEX DNA-seq Kit (Rubicon Genomics). Quality and quantity of libraries were assessed on 2100 Bioanalyzer using HS DNA Kit (Agilent), and on Qubit 2.0 Fluorometer using Qubit dsDNA HS Assay Kit (Life Technologies). The libraries were sequenced (2 × 75 bp) on Illumina HiSeq2500 and HiSeq4000 platforms, according to our modified experimental and software protocols for generation of high-quality data for low-diversity samples^16^.

### Primary i-BLESS data analysis

Data analysis and interpretation were performed using our *iSeq* software for multi-scale analysis and high-level interpretation of DSB sequencing data, and are described in detail elsewhere (http://breakome.utmb.edu/software.html). Briefly, we used *iSeq* to ensure sequencing data quality before mapping. Next, *iSeq* was used to remove proximal and distal i-BLESS barcodes (TCGAGGTAGTA and TCGAGACGACG, respectively). Reads labeled with the proximal barcode, which are directly adjacent to DSBs, were selected and mapped to the yeast genome S288C with bowtie v0.12.2 using alignment parameters ‘-m1 -v1’ to exclude multiple mapping and low-quality reads. The tail base pairs were trimmed using bowtie ‘-3’ option, the parameter choice was based on the *iSeq* quality report. Hygestat_BLESS v1.2.3 (part of *iSeq* software suite, available from http://breakome.eu/software.html) was used to implement mappability correction and identify DSB-rich regions (or “fragile regions”) with a significant increase of read numbers (hypergeometric test with Bonferroni correction, *P* value < 0.001) in treatment versus control samples within windows of selected size.

### Analysis of BamHI, NotI, SrfI, AsiSI and I-SceI data

To analyze samples in which DSBs were induced by a restriction enzyme (BamHI, NotI, SrfI, AsiSI or I-SceI), we used hygestat_bed (part of *iSeq* software suite) to identify reads within a given vicinity of the cutting sites and estimate *P* values of enrichment (hypergeometric test with Bonferroni correction). Absolute DSB frequencies for non-canonical I-SceI sites were estimated using qDSB-seq method^22^ using I-SceI spike-in.

### Autocorrelation and cross-correlation analysis

To identify potential periodic patterns in mapped i-BLESS reads, we performed autocorrelation analysis of the i-BLESS data, using 1 nt bin size and 800 nt range. To further study localization of i-BLESS reads, we computed cross-correlation of the i-BLESS data with the MNase-seq^18^ data on nucleosome positioning. The cross-correlation was calculated for every integer shift value *n* by determining Pearson correlation coefficients between i-BLESS read depth at all genomic positions and MNase read depth, shifted by *n* nucleotides. To compute autocorrelation for i-BLESS data, we cross-correlated it with itself.

### Break-seq data analysis

We mapped Break-seq reads (HU 1 h, GSM1419918) to the budding yeast genome sequence and obtained read depth of DSBs using the same parameters as for analysis of the i-BLESS data, described above.

### G-quadruplex analysis

We used AllQuads^29^ to identify G4 structures. Both canonical intra-strand and non-canonical inter-strand G4 structures were predicted, and then annotated according to *Saccharomyces* Genome Database^41^. To test whether the significantly fragile intervals (see Primary i-BLESS data analysis) were enriched in G4 sequences, enrichment analysis was performed as described below (Enrichment analysis) using intervals of mappable length of 20 nucleotides. To compare G4s and other regions, we defined inside and flanking regions of G4s. The flank of a given G4 region was defined as flanking regions with the same total length as the G4 sequence on both sides. G4 sequences were clustered based on the loop length into: 3 subcategories (G4 L_1–4_: all loops ≤ 4 nt; G4 L_5–7_: all loops ≤ 7 nt, but at least one loop > 4 nt; G4 L_8–16_: all loops ≤ 16 nt, but at least one loop > 7 nt) or 16 groups G4 L_k_, each group consisting of G4 with all loops of length ≤ *k*, but at least one loop of length = *k*. The number of i-BLESS reads was counted using in-house PERL script. Kolmogorov–Smirnov test was used to evaluate the significance of the differences between them. Absolute DSB frequencies near G4 structures were estimated using qDSB-seq method^22^ with NotI spike-in.

### Enrichment analysis

To determine whether a given genomic feature is enriched in DSB-rich regions, we used hygestat_annotations v. 2.0 (part of iSeq software suite). Specifically, hygestat_annotations computed the proportion of mappable nucleotides belonging to both the DSB-rich regions and the given feature (observed overlap), and the proportion of mappable nucleotides belonging to both genomic regions and the given feature (expected overlap). Next, we performed 100–1000 permutations of DSB-rich region assignments among the windows considered and used them to calculate the empirical distribution of the ratio under the null hypothesis that the given feature and DSB-rich regions are independently distributed in the yeast genome. We used this distribution to estimate the *P* value for the feature enrichment (ratio of observed to expected overlap > 1) or depletion (ratio of observed to expected overlap < 1) inside DSB-rich regions. In the enrichment analysis, i-BLESS data was annotated to multiple features, including the predicted G4s and gene promoters.

### Code availability

The code used to generate results reported in this paper is available from http://breakome.utmb.edu/software.html upon request.

## Electronic supplementary material

Supplemental Material

## Data Availability

The data reported in this paper are deposited to the NCBI Sequence Read Archive (SRA), accession code SRP125409.
