# Supplementary material for: i-BLESS is an ultra-sensitive method for detection of DNA double-strand breaks
Source: Commun Biol. 2018 Oct 31;1:181. doi: 10.1038/s42003-018-0165-9 (PMC6208412; doi:10.1038/s42003-018-0165-9)
Supplement: Supplementary file 1 — Supplemental Material [file 42003_2018_165_MOESM1_ESM.pdf]

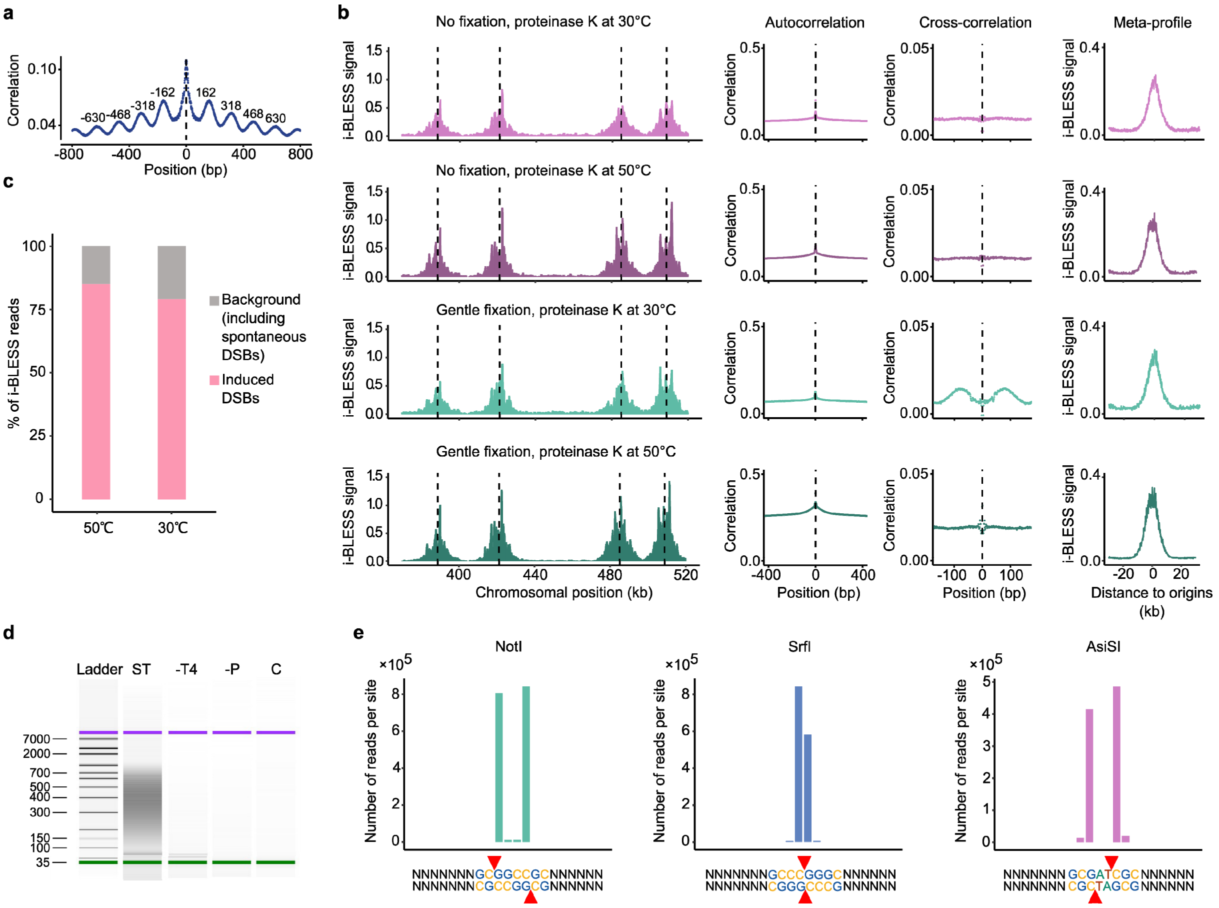

Supplementary Figure 1. i-BLESS method optimization and validation. (**a**) Autocorrelation pattern of i-BLESS signal characteristic for i-BLESS data exhibiting low signal-to-noise ratio. Periodicity of the signal (~162 bp) corresponds to the typical distance between nucleosomes in *S. cerevisiae* (162 ± 6 bp). (**b**) Impact of experimental protocol parameters on quality of i-BLESS data. *mec1-1 sml1-1* cells treated with hydroxyurea were not fixed or subjected to gentle fixation (2% formaldehyde for 5 min) and treated with 50 µg mL^{-1} proteinase K O/N at 30 or 50ºC. For each sample, i-BLESS signal around replication origins (dotted vertical lines) in a representative region of chromosome VII, autocorrelation of i-BLESS signal, cross-correlation of i-BLESS data with MNase data and averaged i-BLESS signal around replication origins are shown. Reads were normalized to 1 million total reads. (**c**) Percentage of reads at NotI, SrfI and AsiSI recognition sites ("Induced DSBs") and rest of the genome ("Background (including spontaneous DSBs)") in wt cells treated with NotI, SrfI and AsiSI enzymes. (**d**) Bioanalyzer 2100 data of PCR products obtained using standard i-BLESS protocol (ST) and protocol without addition of T4 ligase (-T4) or proximal adapter (-P) during DSB labeling. C corresponds to non-template control. (**e**) Averaged i-BLESS signal in a 22 bp window around NotI (left), SrfI (middle) and AsiSI (right) cutting sites (marked with red arrows) for wt cells treated with all 3 enzymes simultaneously.

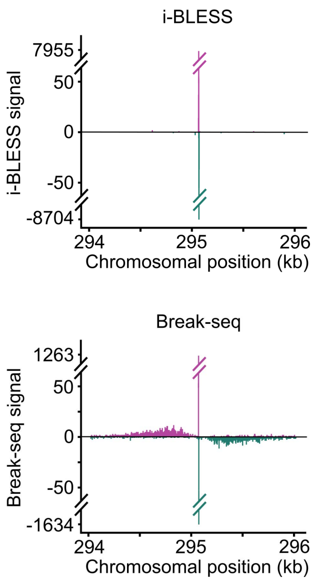

Supplementary Figure 2. i-BLESS and Break-seq signals around selected BamHI recognition site localized on chromosome II. Presented data is representative for all BamHI sites.

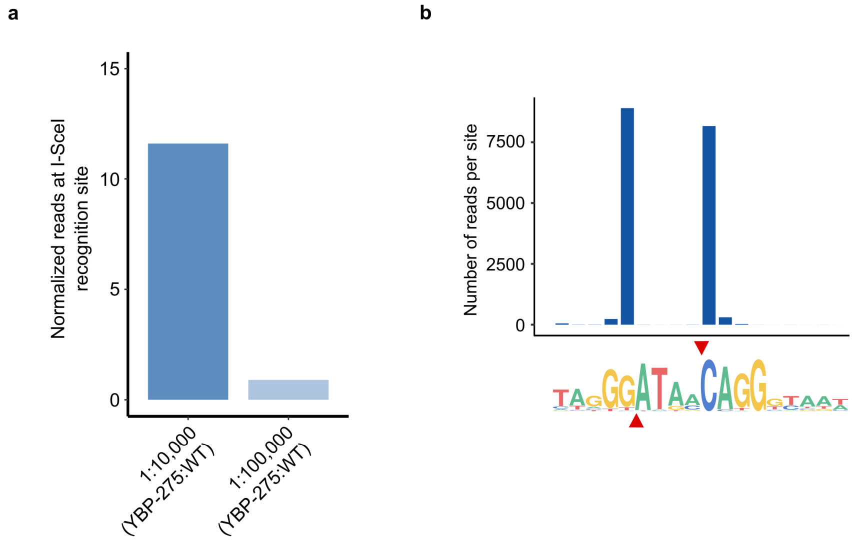

Supplementary Figure 3. Sensitivity of i-BLESS. (**a**) Number of reads within a 16 bp window surrounding the I-SceI recognition site in 1:10,000 and 1:100,000 diluted samples (YBP-275:wt cells), normalized to the total number of mapped reads. (**b**) Averaged i-BLESS signal in a 18 bp window around non-canonical I-SceI recognition sites. I-SceI motif was created for 24 non-canonical I-SceI sequences detected in *in vitro* I-SceI treated wt and YBP-275 cells (mixed at proportion 10,000:1) using WebLogo. The overall height of the stack indicates the sequence conservation at given position, while the height of nucleotides indicates the relative frequency of each nucleotide at that position.

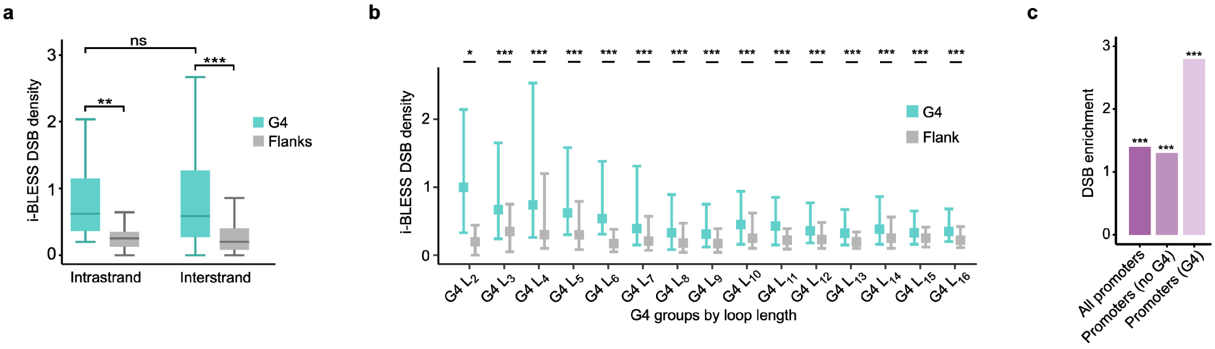

Supplementary Figure 4. G4-related genome instability. (**a**) DSB densities inside and outside G4 sequences forming intra- or interstrand G4s. DSB density was defined as a number of i-BLESS reads mapped to a given region, divided by region length. "G4" are canonical G-quadruplex structures identified by AllQuad software (see Methods). "Flanks" are their left and right adjacent regions half of the length of their corresponding G4. Median (center line), lower/upper quartiles (box limits), and lower/upper adjacent (whiskers) are shown. *P* values were calculated by two-sided Kolmogorov-Smirnov test, *** $P < 0.001$, ** $P < 0.01$, ns – not significant. (**b**) DSB densities inside and outside of G4s containing loops of the indicated length. G4 sequences are classified into 16 groups G4 L_k, each group consisting of G4s with all loops of length $\leq k$, but at least one loop of length $= k$. Group G4 L_1, which was too small for statistical analysis (n=4), was omitted. Median and lower/upper quartiles are shown. *P* values were calculated by Wilcoxon signed-rank test, *** $P < 0.001$, * $P < 0.05$. (**c**) DSB enrichment within all promoters (n=5113), non-G4 promoters (n=5034) and promoters containing G4s (n=75). *P* values were calculated by permutation test, *** $P < 0.001$.

Supplementary Table 1. Analysis of I-SceI cassette position in the genome

Reads position	Reads number
Both reads mapped to the cassette	11,916
One read within 500 bp from cassette end and paired read at chromosome VII (ADH4 locus)	589
One read within 500 bp from cassette end and paired read within 500 bp from cassette start (indicating multiple adjacent cassettes)	0
One read within 500 bp from cassette end and paired read at other genomic locations:	
Chr I	2
Chr II	5
Chr III	1
Chr IV	12
Chr V	5
Chr VI	2
Chr VII	6
Chr VIII	2
Chr IX	1
Chr X	2
Chr XI	2
Chr XII	9
Chr XIII	7
Chr XIV	8
Chr XV	7
Chr XVI	2
Chr M	6

Paired end sequencing of gDNA isolated from YBP-275 cells was performed, reads that mapped to I-SceI cassette were identified and position of a corresponding read was analyzed.

Supplementary Table 2. Non-canonical I-SceI recognition sites identified by i-BLESS

Chromosome	Position	Sequence	Estimated percentage of cells with DSB at given site
II	545747-545762	TTCGGATTACAGGGCAGA	0.03
IV	109853-109868	TAGGCATACCAGGTTGTA	0.94
IV	842855-842870	TAGGGATGGCAGGCTATT	0.03
IV	1247343-1247358	AAGGGATAACAGGTTTAT	4.80
V	511664-511679	CCGGTATGCCAGGTCAAT	0.05
V	543600-543615	TTTTGATAACAGGCTAAA	0.02
VI	35092-35107	TATGGATAGCAGGGTATC	0.07
VII	139222-139237	TTGGGATAACAGGATCAC	0.01
VII	239021-239036	ATGGGATAACATGGCAAA	0.10
VIII	103663-103678	CAGGGATAACATGTCTTC	0.01
X	205102-205117	CAGGGATACCAGGTCTGA	8.29
X	231973-231988	TCTGGATTCCAGGTTAAT	0.18
X	598264-598279	TAGTGATGCCAGGGTCAA	0.10
XI	407927-407942	TACGTATAACAGGGTCGA	0.09
XII	271196-271211	TAAGGAAGCCAGGGCAAT	0.63
XII	445243-445258	TAGGGATAACAGGGGCGA	6.90
XII	886994-887009	AAAGGATAACAGGTTATT	1.27
XII	925062-925077	TAGGGATGACAGGTAGAA	1.07
XIV	46309-46324	TTCGGATGCCAGGGCTTA	0.05
XV	448024-448039	GAGGTTCATCAGGATAAC	0.06
XV	593515-593530	TAATGATACCAGGGCAGT	0.04
XV	825678-825693	TTGGGATAACCAGGCACA	0.02
XVI	652804-652819	CAAGGATAACAGGGGATA	0.01
M	34507-34522	TTTGGATACCAGGCCAAA	0.57

Supplementary Table 3. Yeast strains used in this study

Strain	Genotype
YBP-275	*MATa-inc, bar1Δ, ade2-1, can1-100, leu2::SFA1, trp1-1, ura3-1, lys2::GAL1p-ISCEI, adh4::URA3::GAL1p::leu2Δ3'::ACT1iΔ3'::IsceIsite, his3::HYG:HOsite::ACT1-iΔ5'::leu2Δ5'*
wt	*MATa, ade2-1, trp1-1, can1-100, leu2-3,112, his3-11,15, ura3, GAL, psi+, RAD5, URA3::GPD-TK(7x)*
mec1-1 sml1-1	*MATa, ade2-1, trp1-1, can1-100, leu2-3,112, his3-11,15, GAL, psi+, ura3::URA3/GPD-TK(7x), mec1-1, sml1-1, RAD5*
pif1-m2	*MATa, ade2-1, ura3-1, his3-11,15, leu2-3, 112, trp1-1, CAN1, GAL, PSI+, sml1:TRP1, pif1-m2*

Supplementary Table 4. Oligonucleotides used in this study

**Name**	**Sequence (5’ to 3’)**
Proximal adapter	[Phos]TACTACCTCGAGAGTTACGCTAGGGATAACAGGGTAATATAGTTT[BtndT]TTTCTATATTACCCTGTTATCCCTAGCGTAACTCTCGAGGTAGTA
Distal adapter	[Phos]CGTCGTCTCGAGAGTTACGCTAGGGATAACAGGGTAATATAGTTTTTTTCTATATTACCCTGTTATCCCTAGCGTAACTCTCGAGACGACG
PCR primer 1	CCCTAGCGTAACTCTCGAGGTAGTA
PCR primer 2	CTAGCGTAACTCTCGAGACGACG
